# Relationships between adaptive and neutral genetic diversity and ecological structure and functioning: a meta-analysis

**DOI:** 10.1111/1365-2745.12240

**Published:** 2014-06-23

**Authors:** Raj Whitlock

**Affiliations:** Institute of Integrative Biology, University of LiverpoolLiverpool, L69 7ZB, UK

**Keywords:** Bayesian mixed-effects meta-analysis, community genetics, ecogenomics, ecological genetics, ecosystem function, genotypic diversity, productivity, species diversity, species richness

## Abstract

1. Understanding the effects of intraspecific genetic diversity on the structure and functioning of ecological communities is a fundamentally important part of evolutionary ecology and may also have conservation relevance in identifying the situations in which genetic diversity coincides with species-level diversity.
2. Early studies within this field documented positive relationships between genetic diversity and ecological structure, but recent studies have challenged these findings. Conceptual synthesis has been hampered because studies have used different measures of intraspecific variation (phenotypically adaptive vs. neutral) and have considered different measures of ecological structure in different ecological and spatial contexts. The aim of this study is to strengthen conceptual understanding by providing an empirical synthesis quantifying the relationship between genetic diversity and ecological structure.
3. Here, I present a meta-analysis of the relationship between genetic diversity within plant populations and the structure and functioning of associated ecological communities (including 423 effect sizes from 70 studies). I used Bayesian meta-analyses to examine (i) the strength and direction of this relationship, (ii) the extent to which phenotypically adaptive and neutral (molecular) measures of diversity differ in their association with ecological structure and (iii) variation in outcomes among different measures of ecological structure and in different ecological contexts.
4. Effect sizes measuring the relationship between adaptive diversity (genotypic richness) and both community- and ecosystem-level ecological responses were small, but significantly positive. These associations were supported by genetic effects on species richness and productivity, respectively.
5. There was no overall association between neutral genetic diversity and measures of ecological structure, but a positive correlation was observed under a limited set of demographic conditions. These results suggest that adaptive and neutral genetic diversity should not be treated as ecologically equivalent measures of intraspecific variation.
6. *Synthesis*. This study advances the debate over whether relationships between genetic diversity and ecological structure are either simply positive or negative, by showing how the strength and direction of these relationships changes with different measures of diversity and in different ecological contexts. The results provide a solid foundation for assessing when and where an expanded synthesis between ecology and genetics will be most fruitful.

## Introduction

A growing number of studies provide evidence that intraspecific genetic diversity can influence the diversity, structure and functioning of plant and animal communities and ecosystems (hereafter ‘ecological structure’; Vellend & Geber 2005; Hughes *et al*. 2008; Whitham *et al*. 2012). This work has been motivated by the realization that evolutionary and ecological processes can take place on the same time-scales and can be interconnected (Antonovics 1976). Understanding the relationship between genetic diversity and higher-order ecological structure is important for several reasons. First, ecological models have often treated individuals within species as ecologically equivalent (assuming limited effects of genetic diversity e.g. Grime 1973; Chesson & Warner 1981; Tilman 1994). To test the validity of this simplifying assumption, we must understand the effects of genetic diversity as a structuring force in communities and ecosystems. Secondly, genetic diversity may modify the responses of communities and ecosystems to anthropogenic environmental change, through responses to selection and adaptation (de Mazancourt, Johnson & Barraclough 2008; Norberg *et al*. 2012). An understanding of the ecological effects of genetic diversity will allow us to anticipate shifts in community structure and function that may occur as correlated responses with population-level adaptation to environmental change (Reusch *et al*. 2005; Sthultz, Gehring & Whitham 2009). Thirdly, the relationship between genetic diversity and ecological structure and function is relevant to restoration ecology and informs on whether restored habitat should be created with a mix of genotypes of each component species (e.g. Vergeer, Sonderen & Ouborg 2004; Broadhurst *et al*. 2008). Finally, these ‘community-genetic’ effects are relevant to agriculture because crop genetic diversity may enhance resistance to arthropod herbivores and increase crop yields (Tooker & Frank 2012).

Studies investigating the relationship between intraspecific diversity and ecological structure (hereafter community genetics studies) have focussed on ecological effects stemming from two levels of genetic organization. One set of studies has documented ecological responses to or associations with genetic diversity itself (e.g. Odat, Jetschke & Hellwig 2004; Crutsinger *et al*. 2006). These studies are the focus of this review. They measure ecological structure and processes relative to the genetic diversity of populations of a focal species, where the focal species’ populations comprise more than one individual. A second, overlapping set of studies has taken an individual-level perspective, in which the objective is to understand whether the genes of individuals of a focal species (e.g. a forest tree) select for particular associated or dependent communities (e.g. an arthropod community; Whitham *et al*. 2003). These studies describe genetic variation of ecological responses among individuals of the focal species and are not considered further here.

Community genetics studies have considered both adaptive and neutral genetic diversity. Adaptive variants influence the phenotype and fitness of the organisms that carry them; neutral variants, on the other hand, are selectively neutral (full definitions are given in Supporting Information Data S1). In this paper, I will treat all intraspecific phenotypic differentiation that has a demonstrable genetic component as ‘adaptive’ (e.g. phenotypic differences among genetically distinct plant clones or ‘genotypes’ observed in a common environment). Thus, neutral genetic variants are defined as being both selectively neutral for the individuals that carry them and ecologically neutral in their effects on other coexisting individuals, species and on ecological processes. Under these definitions, only adaptive genetic diversity can drive selection among coexisting species or respond to selection imposed by the ecological context, and thus, only adaptive genetic diversity has ecological consequences. Neutral diversity, on the other hand, can become associated with ecological structure indirectly, through the location-specific action of genetic drift and migration (Vellend & Geber 2005). Thus, both neutral and adaptive genetic diversity can correlate with ecological structure, but the underlying processes and mechanisms driving these associations differ (Box Box 1 gives an overview of the mechanisms linking genetic diversity and ecological structure; Vellend & Geber 2005; Hughes *et al*. 2008).

**Box 1. Mechanisms connecting genetic diversity and ecological structure**

Neutral genetic diversity can become correlated positively with species diversity when communities exist as demographically isolated patches of different sizes (Vellend 2003; Vellend & Geber 2005). Population size and isolation influence neutral genetic diversity through their effects on genetic drift and immigration. Rates of genetic diversity loss through drift are greater in smaller, more isolated populations, and these often contain less genetic diversity (Frankham 1996, 1997). The balance between species extinction and immigration in communities that are small in size or that are isolated is expected to influence species diversity in a similar way; small islands often have lower species diversity (MacArthur & Wilson 1967; Rosenzweig 1995). If, however, communities have a fixed total size (e.g. number of individuals), then increases in species richness can lead to smaller population sizes for each component species. These decreases in population size increase the likelihood that neutral genetic diversity will be lost from the component populations through genetic drift, leading to negative correlations between intraspecific genetic diversity and species diversity (Vellend 2005).

Adaptive genetic diversity can drive intra-generational or ‘instantaneous’ effects on ecological structure and functioning via two routes. First, genotypic composition can regulate ecological responses via individual-level independent and additive effects of each component genotype (‘additive responses’). In these cases, community- or ecosystem-level responses in genetically diverse mixtures can be predicted from information on the monoculture ‘performance’ of the component genotypes and their population frequencies (Hughes *et al*. 2008). Additive effects include the selection probability effect, or sampling effect, in which mixtures of different genotypes may be more likely to contain at least one genotype contributing an extreme phenotype or ecological response (Huston 1997; Loreau & Hector 2001). Non-additive responses to genetic diversity arise through interactions among coexisting genotypes and occur when genetically diverse mixtures support communities or ecosystem responses that cannot be predicted from knowledge of both genotypic composition and the monoculture performance of component individuals. These responses can be generated by several processes, including niche partitioning (more efficient use of the resource base), facilitation and inhibition effects (Hughes *et al*. 2008).

Associations between adaptive genetic diversity and ecological structure can also arise dynamically, through parallel responses to selection pressures that vary across space or time. These responses incorporate the effects of selection and eco-evolutionary dynamics over time. If the total niche space in a community is fixed, then the realized niche breadth (i.e. adaptive genetic diversity) of individual populations could be reduced as more species compete over the total niche space (cf. Van Valen 1965). This would induce negative relationships between adaptive genetic diversity and ecological structure (Vellend 2005). On the other hand, competition or facilitation occurring within local neighbourhoods dominated by different species or genotypes could provide a mosaic of biotic niches that stimulate diversifying selection within species (Turkington & Harper 1979; Aarssen 1989). In this scenario, greater species diversity would facilitate the maintenance of higher levels of adaptive genetic diversity.

### The need for synthesis

The number of studies investigating the relationship between genetic variation and ecological diversity, structure and functioning has grown rapidly since the publication in 2003 of a clutch of landmark papers (including Booth & Grime 2003; Neuhauser *et al*. 2003; Whitham *et al*. 2003). During 2012, more than 70 papers were published in this broad research area (including studies in both semi-natural and agricultural settings), and this expansion has seen a marked diversification in the types of study published. These now include experimental and observational studies focussing on adaptive and neutral diversity (e.g. Tack & Roslin 2011; Wei & Jiang 2012), studies whose individual replicate populations span a wide range of spatial scales (from 1 m to ∼500 m; He & Lamont 2010; Chang & Smith 2013) and that encompass a wide range of ecological contexts (e.g. studies focussed within and between trophic levels; Hovick, Gumuser & Whitney 2012; Moreira & Mooney 2013). During this rapid expansion phase, narrative reviews have examined the mechanisms through which genetic diversity can modify or become associated with ecological structure (Vellend & Geber 2005; Hughes *et al*. 2008), have considered the circumstances under which genetic diversity is likely to have its greatest effects (e.g. Johnson & Stinchcombe 2007; Hughes *et al*. 2008) and have established high-level frameworks for the integration of community ecology and evolutionary biology (Johnson & Stinchcombe 2007). Several of these reviews have called for a shift in focus towards studies that investigate the particular conditions under which genetic diversity impacts on or becomes associated with ecological structure (Johnson & Stinchcombe 2007; Hughes *et al*. 2008). Thus, there is now a need for a quantitative synthesis of the available evidence, in order to connect the growing literature to mechanistic frameworks and to predictions that have been set out in narrative reviews and to assess the extent of heterogeneity in genetic effects on ecological structure.

I argue that the need for synthesis is strong in two specific areas. First, we need to quantify the direction and strength of the relationship between genetic diversity and ecological diversity, structure and functioning. The only published formal meta-analysis in this subject area has measured the strength of these effects, but not their direction, and also focussed only on the effects of adaptive genetic diversity in a limited number of studies (Bailey *et al*. 2009). This study found that the effects of genetic diversity within plant species were greatest on the plants’ own phenotypes (e.g. production of leaf secondary metabolites, physiology) and had successively weaker effects on community-level and ecosystem-level responses (e.g. species diversity, nutrient cycling). Information on the expected direction of the relationship between genetic diversity and ecological level structure would create a benchmark for comparison within the community genetics literature and may also be important within a conservation context (Bangert *et al*. 2005). Secondly, we need to understand whether neutral and adaptive genetic diversity show similar correlations with ecological structure. In other words, can neutral and adaptive diversity be used as ecologically equivalent measures of genetic variation? Early studies in the community genetics literature documented positive correlations between adaptive genetic diversity and measures of community structure (e.g. Booth & Grime 2003; Johnson, Lajeunesse & Agrawal 2006). More recently, studies employing neutral genetic diversity have been used to challenge the hypothesis that genetic diversity and ecological structure are positively correlated (Silvertown, Biss & Freeland 2009; Taberlet *et al*. 2012). This approach to testing the relationship between genetic diversity and ecological structure assumes that neutral and adaptive genetic diversity are correlated or interchangeable measures of variation. However, this assumption may not be valid, because neutral and adaptive variations are connected with ecological structure via different mechanisms (Box Box 1; Vellend & Geber 2005) and often show a poor correlation with each other (Reed & Frankham 2001). Quantitative synthesis is needed to determine whether these measures of genetic diversity associate similarly with ecological structure. If they do, then these measures of diversity can be used interchangeably. If they do not, then they cannot.

In this review, I used meta-analysis to determine the strength and direction of the relationship between genetic diversity within plant populations, and community-level and ecosystem-level measures of ecological structure. I also investigated the extent to which this relationship differs for neutral and adaptive measures of genetic diversity, and with other aspects of the ecological context. I show that levels of adaptive genotypic diversity are positively, but weakly associated with measures of both community structure and ecosystem functioning but that there is no consistent relationship between neutral genetic diversity and ecological structure.

## Materials and methods

### Literature searches

On 12 August 2013, I conducted literature searches to identify studies that investigated the relationship between within-population genetic diversity and community structure, diversity and ecosystem functioning. Literature searches interrogated three online repositories: Web of Knowledge, Science Direct and Scopus (search terms and structure are given in Table S1). Data base-specific literature searches were merged in Endnote (version X4.0.2), and duplicate records were discarded to give a master data base containing 8670 articles. These articles were filtered to remove non-journal and irrelevant clinical and biomedical articles from the data base, leaving 6980 articles (Data S2; Table S2).

### Review scope and inclusion criteria

This review synthesized results from experimental or field-based empirical studies. Theoretical papers were excluded from the review, as were simulation studies and other reviews. Reviews focusing on the relationship between genetic diversity and ecological structure were retained as relevant, and their bibliographies were checked against the Endnote data base to identify any further relevant articles from the primary literature.

This review focussed on communities or ecosystems containing a population of at least one plant species whose genetic diversity had either been manipulated, or had been measured. Both natural and agricultural or farmed communities and ecosystems were valid subjects. As the aim of this review was to synthesize the effects of population-level genetic diversity, I excluded studies that dealt only with community- and ecosystem-level responses to genotypic or genetic identity. In other words, the plants whose genetic diversity were measured or manipulated must have comprised genuine ‘populations’ containing more than one individual.

I refer to the species whose genetic diversity was observed as the ‘focal species’, and I define the spatial area within which a focal population resides and for which ecological outcome measures were recorded, as the sampling unit. The review included studies that manipulated genetic diversity experimentally and that observed non-manipulated genetic diversity of focal populations using molecular markers. Both neutral genetic diversity (measured with molecular markers) and adaptive phenotypic diversity were accepted as valid measures of genetic diversity (the ‘exposure variable’; Table 1). For diversity to qualify as ‘adaptive’, there must have been evidence that the phenotypic variation was genetic in nature, for example, individuals were raised in a common environment, or where a phenotype had a known genetic basis or heritability, or where marker studies identified individuals carrying different phenotypes as being genetically distinct. Well-documented natural ecotypes from widely separated populations (e.g. *Arabidopsis thaliana*) and agricultural cultivars were considered to meet this condition as well. Other studies not meeting this criterion were excluded. Studies focusing exclusively on community and ecosystem responses to intra-genomic diversity, for example, arising from interspecific hybridization, inter-population outcrossing or inbreeding were also excluded, because they investigate ecological responses to genetic identity rather than to genetic diversity measured at the population level.

**Table 1:** Measures of neutral and adaptive genetic diversity and ecological structure used in this review. Outcome types used in Fig. 1 are given in bold

Variable	Variable type	Valid measures
Neutral genetic diversity	Exposure	Expected heterozygosity *H*_E_, allelic richness *A*_R_, inter-individual genomic dissimilarity, marker polymorphism (these measures were restricted to variation at molecular marker loci that is presumed to be selectively neutral)
Adaptive genetic diversity	Exposure	Numbers of clones (genotypic richness), ecotypes, cultivars, genetically distinct individuals or families of related individuals
Community structure	Outcome	Species **richness**, species **evenness** (the extent to which the abundances of coexisting species are equal), species **diversity** (a measure of the effective number of species, incorporating both species richness and evenness), species compositional dissimilarity (between-community differences in species abundance structure, often assessed using **ordination** techniques)
Ecosystem function	Outcome	**Productivity** (biomass production, photosynthetic rate), **‘stocks’** (levels or availability of elements and nutrients, and non-living biomass pools), **‘flux’** (changes, or rates of change of stocks) and **‘stability’** (measures of ecosystem resistance or resilience to environmental perturbation).

### Relevant outcomes

Relevant outcomes were observations of ecological structure: either community- or ecosystem-level diversity, structure and functioning recorded within a community or ecosystem containing a focal species’ population (Table 1). I considered only community-level outcomes that were multi-species in nature, that is, outcome responses for individual species were excluded (including abundance measurements and reproductive success specific to a single species). Ecosystem-level outcomes were stocks and flows of elements, nutrients or energy, and measures of ecosystem resistance and resilience to environmental perturbation (Table 1). Ecological outcomes were only valid when they were observed in an area that corresponded with the sampling unit (containing the population of the focal species whose genetic diversity was measured or manipulated). I excluded several studies for which it was unclear whether focal plant populations and associated communities were spatially coincident (Wendel & Percy 1990; Borgen 1996; Taberlet *et al*. 2012). Ecosystem-level outcomes were accepted even if they applied only to a single species, usually the focal species (e.g. a canopy-dominant plant).

### Article assessment procedure

I assessed article relevance using a three-stage procedure, in which first article titles, then article abstracts and finally article full texts were assessed against the review scope as defined above (full details are given in Data S3). The repeatability of the title and abstract assessments were tested through independent assessment of a random subset of 517 articles (title assessment) and 100 articles (abstract assessment; second assessment was undertaken by S. Trinder). The level of concordance in assessment was measured using the Kappa coefficient of agreement (titles, к = 0.625, excellent agreement; abstracts, к = 0.550, moderate agreement). I did not check the objectivity of full-text assessment using Kappa analyses as I had done for title and abstract assessment. However, during full-text assessment, I was able to compare a complete description of the methods employed in each study with the review inclusion criteria given above, leading to robust decisions on study relevance. It should be noted that this review is not intended to be exhaustive in its scope. Although I have made every effort to identify and collate the relevant evidence, it is possible that some relevant papers are missing from this review.

### Sources of heterogeneity

Potential sources of heterogeneity were extracted from relevant articles for use as explanatory variables in meta-analyses. Focal species identity and exposure type (whether or not genetic diversity was neutral or adaptive; Table 1) were recorded for each study. Ecological outcomes were categorized in two ways. First, the *outcome level* was recorded – whether outcomes were at the community level or the ecosystem level. Secondly, *outcome type* was recorded; this was a categorical description of the outcome variable nested within outcome level (e.g. species diversity, richness or evenness; Table 1). Different measures of genetic diversity were categorized into different *genetic diversity type*s (Table 1). The outcome and diversity types listed in Table 1 represent the variables currently in use in the community genetics literature and were not used to prescribe the scope of the review. For the studies that manipulated focal species’ genotypic diversity experimentally, I calculated different effect sizes for each possible pairwise comparison of genetic diversity levels (see effect size calculation, below). In each case, I recorded the size of the genotypic diversity manipulation as the difference in genotypic diversity between the levels contrasted to construct each effect size (*intervention size*). My rationale for this was that larger effect sizes could be associated with greater differences in genetic diversity. For the studies that reported outcomes at the community level, I recorded a variable called *trophic contrast* that described whether focal species were at the same trophic level as the responding community (intra-trophic contrast) or at a different trophic level (inter-trophic contrast). Study sampling designs for the focal species (*sampling strategy*) were documented as either within-population, or between-population, or cultivar. The latter category was applied where the focal individuals were from agricultural cultivars or breeds that did not belong to any natural population. The sampling strategy variable for studies that manipulated focal species genetic diversity described whether the individuals of the focal species had been collected from different populations or from within the same population. The corresponding variable for observational studies described whether sampling units were focal species’ populations or were plots or stands within a single contiguous focal species’ population. I recorded the breadth of sampling units in metres, as a measure of their size (*sample unit scale*). Where studies provided information on the area or volume of the sampling unit, I used the square- or cube-root of this value to approximate sample unit breadth; the median sample scale was used where the sample units within a study varied in area. The overall spatial extent of a study (*study scale*) was the Euclidian distance between the most widely separated sampling points for focal species’ individuals (for studies manipulating adaptive genetic diversity) or populations (for studies investigating natural gradients of neutral genetic diversity). To investigate whether the difference between study scale and sample unit scale could influence community-genetic effect sizes (Tack, Johnson & Roslin 2012), I recorded the *disparity in sampling scale* as log (study scale in km/sample unit scale in km). This variable was extracted only for studies where genetic diversity had been manipulated. The final source of heterogeneity that I considered was intended to investigate the effects of locality characteristics on associations between neutral genetic diversity and ecological structure (Box Box 1; Vellend & Geber 2005). First, I noted when the sample unit area was constrained so that focal species population size co-varied negatively with the size or extent of the rest of the community (*area constraint*). Secondly, I noted when sample units within studies were both variable in area and were isolated from other sample units, representing effective demographic islands (*island effect*).

### Data extraction and effect size calculation

Data were extracted from 69 articles (70 studies) to calculate effect sizes (hereafter, community-genetic effect sizes) relating within-population genetic diversity to measures of ecological structure. I extracted data from article text and from article figures using ImageJ image analysis software (version 1.45s). When the relevant data were not presented in an extractable form, the article authors were approached in order to gain access to the necessary data summaries. Authors were also approached to gain missing information on the sources of heterogeneity. When relevant data were presented as a time series of repeated measures, I used data from the final time point to calculate effect sizes.

Effect sizes were calculated in two ways, depending on whether studies focused on neutral genetic diversity within natural populations or whether they focused on experiments where the focal species’ adaptive genetic diversity had been manipulated. Thus, there were two effect-size data sets, one for each of these study designs. Effect sizes for neutral diversity were calculated from correlation coefficients that compared population-level genetic diversity with community structure or ecosystem functioning. Community-genetic effect sizes were calculated by applying Fisher's *z*-transformation to the raw correlation coefficients (these effect sizes are referred to as *z*(*r*) statistics). The measurement error variance (*mev*) of these effect sizes was calculated as 1/(*n* − 3), where *n* is the number of populations (Borenstein *et al*. 2009). I used the standardized mean difference *d* as the measure of effect size in studies that manipulated focal species’ genetic diversity experimentally (Borenstein *et al*. 2009). This measure of effect size expresses the difference between two means in units of standard deviations (Borenstein *et al*. 2009). The variance of *d* (*mev*) was calculated as ((*n*_1_ + *n*_2_/*n*_1_ * *n*_2_) + *d*^2^/(2* (*n*_1_ + *n*_2_ − 2))) * ((*n*_1_ + *n*_2_)/(*n*_1_ + *n*_2_ − 2)). I used this measure of effect size for experimental studies because many of these studies presented effects for only two levels of genetic diversity; it would not have been possible to fit a correlation to these data. Studies manipulating focal species’ genetic diversity usually presented data for outcome variables (e.g. species richness) as means and standard errors grouped by genetic diversity level (e.g. the number of distinct clones represented in an experimental community). Means, standard errors and sample sizes were extracted for each outcome variable for each level of focal species’ genetic diversity. These were then used to calculate the standardized mean difference for each unique pairwise combination of genetic diversity levels. For example, a study that created experimental communities with one, three and six clones of the focal species would yield three effect sizes (contrasting ecological responses for three vs. one, six vs. one and six vs. three genotypes per community). I calculated *d* so that effect sizes would be positive when species diversity increased with genetic diversity, or when productivity, nutrient and elemental stocks increased with genetic diversity, or when fluxes or rates of change of the stocks decreased with increasing genetic diversity (I viewed nutrient or stock retention as a ‘positive’ outcome).

### Statistical analyses

Meta-analyses synthesizing the effect sizes were conducted using Bayesian mixed-effects meta-analytic models (function MCMCglmm within the MCMCglmm package in R Development Core Team 2008; Hadfield 2010; Hadfield & Nakagawa 2010). Standardized mean difference effect sizes (*d*) and effect sizes based on correlation coefficients (*z*(*r*)) were analysed separately. To take account of effect size precision, I fitted measurement error variance values (*mev*) about the effect sizes as a set of variance components. I assumed these to be known without error and they were fixed in the analysis, rather than estimated (Hadfield & Nakagawa 2010). To model the dependence of effect sizes within studies, I fitted study identities as random effects (multiple effect sizes collected from individual studies can be considered to be a series of non-independent repeated measures). The sources of heterogeneity (explanatory variables) were fitted as fixed effects. I fitted both categorical and continuous sources of heterogeneity individually (i.e. one predictor variable in each model). This was because imbalance meant that some pairs of these variables had combinations of factor levels containing no effect sizes. For each of these models, I excluded effect sizes where the corresponding source of heterogeneity (predictor) contained unknown or missing information. Three studies within the *z*(*r*) (correlative) data set used molecular markers to infer numbers of clones within sampling units and therefore contributed putatively adaptive community-genetic effect sizes. Heterogeneity between putatively adaptive and neutral effect sizes in this data set was assessed, and then, the adaptive effect sizes were removed to allow analyses that focussed on neutral genetic diversity. Effect sizes *z*(*r*) for these adaptive measures of diversity did not differ significantly from the remaining correlative effect sizes based on neutral diversity (*pMCMC* = 0.952).

MCMC chains were run with a burn-in of 10^4^ iterations, and then for a further 10^6^ iterations, during which parameters were extracted from the chain at a thin interval of 1000 iterations. It was necessary to adapt the number of burn-in and sampling iterations for two of the models to allow them to run. I used non-informative uniform improper distributions on the standard deviation of random effects as the priors for variance components (Gelman 2006). Default settings were used for priors for the explanatory variables. Starting values for variance components were drawn from a half-normal distribution with mean 0.798 and variance 0.363, and these starting values were over-dispersed relative to the posterior distribution. I assessed convergence of MCMC chains using Gelman–Rubin diagnostics applied to chains from three replicate runs of each model (Gelman & Rubin 1992). The extent of chain mixing was assessed using autocorrelation analysis, effective size statistics and by plotting and inspecting MCMC samples. Pooled effects were estimated for the ‘average’ study (i.e. at the intercept of the study random effects) and were extracted from models as posterior means with 95% credible intervals. The point estimates for pooled effects were considered to be statistically consistent (‘significant’) when their corresponding credible intervals did not overlap with zero.

Heterogeneity in community-genetic effect sizes attributable to different hierarchical levels in the meta-analyses was assessed using the approach presented by Sutton *et al*. (2011); heterogeneity for each variance component was expressed as a proportion of variance relative to the total variance. This method required an estimate of the ‘typical’ measurement error variance, for which I used the median *mev* (Whitlock *et al*. 2013).

A known limitation of meta-analyses is that when the literature suffers from publication bias, then pooled (average) effect sizes can also be biased. For example, in the community genetics literature, we might expect that studies would be more publishable if they reported positive relationships between genetic diversity and community-level diversity (assuming a ‘diversity begets diversity’ relationship). Non-significant negative effects observed in studies with small sample sizes would be more likely to be relegated to the file drawer. Such bias would result in asymmetry in the distribution of effect sizes around the pooled effect size, and this asymmetry can be detected visually using forest plots (Borenstein *et al*. 2009). I checked for publication bias within the effect size data sets using enhanced funnel plots (implemented in the R package Metafor; Viechtbauer 2010).

## Results

I reviewed 69 studies reporting relationships between genetic diversity and community diversity, structure and ecosystem function published between 1999 and 2013 (Table 2). From these, I extracted a total of 423 community-genetic effect sizes. I observed an overall positive relationship between adaptive measures of genetic diversity and ecological responses (*d *=* *0.178; 95% credible interval 0.063–0.302; *pMCMC* = 0.002). The correlation between neutral genetic diversity and ecological structure was not consistently different from zero (*z*(*r*) = 0.139; 95% credible interval -0.024–0.311; *pMCMC* = 0.110).

**Table 2:** Species and studies included in the review. Morphotype describes the morphological form of the plant focal species. Exposure type indicates whether an adaptive or neutral measure of genetic diversity of the focal species was used. The effect size measure and outcome levels indicate the type of effect size calculated (standardized mean difference, *d*; *z*-transformed correlation coefficients, *z*(*r*), and the levels at which outcomes were measured (community level or ecosystem level), respectively

Focal species	Morpho-type	Exposure type	Effect size meas-ure	Outcome levels	Citations
*Ammophila breviligulata*	Herb.	Adaptive	*d*	Diversity	Crawford & Rudgers (2013)
*Ammophila breviligulata*	Herb.	Adaptive	*d*	Ecosystem	Crawford & Rudgers (2012)
*Arabidopsis thaliana*	Herb.	Adaptive	*d*	Ecosystem	Crawford & Whitney (2010)
*Arabidopsis thaliana*	Herb.	Adaptive	*d*	Ecosystem	Kotowska, Cahill & Keddie (2010)
*Arabidopsis thaliana*	Herb.	Adaptive	*d*	Ecosystem	Weltzin *et al*. (2003)
*Arabidopsis thaliana*	Herb.	Adaptive	*d*	Ecosystem	Hovick, Gumuser & Whitney (2012)
*Baccharis salicifolia*	Shrub	Adaptive	*d*	Diversity	Moreira & Mooney (2013)
*Brassica oleracea*	Herb.	Adaptive	*d*	Ecosystem	Hamback, Bjorkman & Hopkins (2010)
*Buchloe dactyloides*	Herb.	Adaptive	*d*	Ecosystem	Gruntman & Novoplansky (2004)
*Cakile edulenta*	Herb.	Adaptive	*d*	Ecosystem	Dudley & File (2007)
*Echium vulgare*	Herb.	Adaptive	*d*	Ecosystem	Bischoff, Steinger & Muller-Scharer (2010)
*Festuca arundinacea*	Herb.	Adaptive	*d*	Ecosystem, Diversity	Iqbal, Nelson & McCulley (2013)
*Festuca rubra*	Herb.	Adaptive	*d*	Ecosystem	Munzbergova, Skalova & Hadincova (2009)
*Lolium perenne*	Herb.	Adaptive	*d*	Diversity, Ecosystem	Jones *et al*. (2011)
Many	Herb.	Adaptive	*d*	Ecosystem, Diversity	Fridley & Grime (2010)
Many	Herb.	Adaptive	*d*	Diversity	Johnson *et al*. (2010)
Many	Herb.	Adaptive	*d*	Ecosystem, Diversity	Booth & Grime (2003)
*Oenothera biennis*	Herb.	Adaptive	*d*	Diversity	McArt, Cook-Patton & Thaler (2012)
*Oenothera biennis*	Herb.	Adaptive	*d*	Diversity	Johnson, Lajeunesse & Agrawal (2006)
*Oenothera biennis*	Herb.	Adaptive	*d*	Ecosystem, Diversity	Cook-Patton *et al*. (2011)
*Origanum vulgare*	Herb.	Adaptive	*d*	Ecosystem	Bischoff, Steinger & Muller-Scharer (2010)
*Poa pratensis*	Herb.	Adaptive	*d*	Ecosystem	Vellend, Drummond & Tomimatsu (2010)
*Populus tremuloides*	Tree	Adaptive	*d*	Diversity	Kanaga *et al*. (2009)
*Populus tremuloides*	Tree	Adaptive	*d*	Ecosystem	Madritch, Donaldson & Lindroth (2006)
*Quercus laevis*	Tree	Adaptive	*d*	Ecosystem	Madritch & Hunter (2002)
*Quercus laevis*	Tree	Adaptive	*d*	Ecosystem	Madritch & Hunter (2003)
*Quercus laevis*	Tree	Adaptive	*d*	Ecosystem	Madritch & Hunter (2004)
*Quercus laevis*	Tree	Adaptive	*d*	Ecosystem	Madritch & Hunter (2005)
*Quercus robur*	Tree	Adaptive	*d*	Diversity	Tack & Roslin (2011)
*Solanum lycopersicum*	Herb.	Adaptive	*d*	Ecosystem	Facelli *et al*. (2010)
*Solidago altissima*	Herb.	Adaptive	*d*	Diversity	Genung *et al*. (2010)
*Solidago altissima*	Herb.	Adaptive	*d*	Diversity	Crutsinger *et al*. (2008b)
*Solidago altissima*	Herb.	Adaptive	*d*	Ecosystem	Crutsinger, Sanders & Classen (2009)
*Solidago altissima*	Herb.	Adaptive	*d*	Diversity, Ecosystem	Crutsinger *et al*. (2006)
*Solidago altissima*	Herb.	Adaptive	*d*	Ecosystem, Diversity	Crutsinger *et al*. (2008a)
*Taraxacum officinale*	Herb.	Adaptive	*d*	Ecosystem	Vellend, Drummond & Tomimatsu (2010)
*Triticum aestivum*	Herb.	Adaptive	*d*	Diversity	Chateil *et al*. (2013)
*Triticum aestivum*	Herb.	Adaptive	*d*	Diversity	Zuo, Ma & Shinobu (2008)
*Triticum aestivum*	Herb.	Adaptive	*d*	Ecosystem	Shoffner & Tooker (2013)
*Zostera marina*	Herb.	Adaptive	*d*	Ecosystem	Ehlers, Worm & Reusch (2008)
*Zostera marina*	Herb.	Adaptive	*d*	Ecosystem	Hughes & Stachowicz (2011)
*Zostera marina*	Herb.	Adaptive	*d*	Ecosystem	Reynolds, McGlathery & Waycott (2012)
*Zostera marina*	Herb.	Adaptive	*d*	Ecosystem	Hughes, Best & Stachowicz (2010)
*Zostera marina*	Herb.	Adaptive	*d*	Ecosystem	Reusch *et al*. (2005)
*Zostera marina*	Herb.	Adaptive	*d*	Ecosystem, Diversity	Hughes & Stachowicz (2004)
*Andropogon gerardii*	Herb.	Neutral	*z*(*r*)	Ecosystem	Avolio & Smith (2013)
*Andropogon gerardii*	Herb.	Adaptive, neutral	*z*(*r*)	Diversity	Chang & Smith (2013)
*Andropogon gerardii*	Herb.	Adaptive, neutral	*z*(*r*)	Diversity	Chang & Smith (2012)
*Anthoxanthum odoratum*	Herb.	Neutral	*z*(*r*)	Diversity	Silvertown, Biss & Freeland (2009)
*Anthyllis vulneraria*	Herb.	Neutral	*z*(*r*)	Diversity	Honnay *et al*. (2006)
*Ardisia crenata*	Shrub	Neutral	*z*(*r*)	Diversity	Zeng *et al*. (2012)
*Briza media*	Herb.	Neutral	*z*(*r*)	Diversity	Helm *et al*. (2009)
*Carex curvula*	Herb.	Neutral	*z*(*r*)	Diversity	Puscas, Taberlet & Choler (2008)
*Carex rariflora*	Herb.	Neutral	*z*(*r*)	Diversity	Vellend & Waterway (1999)
*Carex sempervirens*	Herb.	Neutral	*z*(*r*)	Diversity	Yu *et al*. (2009)
*Daviesia triflora*	Herb.	Neutral	*z*(*r*)	Diversity	He & Lamont (2010)
*Euptelea pleiospermum*	Tree	Neutral	*z*(*r*)	Diversity	Wei & Jiang (2012)
*Fagus sylvatica*	Tree	Neutral	*z*(*r*)	Diversity	Wehenkel, Bergmann & Gregorius (2006); Wehenkel, Corral-Rivas & Hernández-Díaz (2011); Bergmann *et al*. (2013)
*Gypsophila fastigiata*	Herb.	Neutral	*z*(*r*)	Diversity	Lonn & Prentice (2002)
*Lolium perenne*	Herb.	Neutral	*z*(*r*)	Diversity	Nestmann *et al*. (2011)
*Maianthemum bifolium*	Herb.	Neutral	*z*(*r*)	Diversity	Honnay *et al*. (1999); Honnay & Bossuyt (2005)
*Picea abies*	Tree	Neutral	*z*(*r*)	Diversity	Wehenkel, Bergmann & Gregorius (2006); Wehenkel, Corral-Rivas & Hernández-Díaz (2011); Bergmann *et al*. (2013)
*Plantago lanceolata*	Herb.	Neutral	*z*(*r*)	Diversity	Odat *et al*. (2010)
*Populus* spp.	Tree	Neutral	*z*(*r*)	Diversity, Ecosystem	Schweitzer *et al*. (2011)
*Populus* spp.	Tree	Neutral	*z*(*r*)	Diversity	Wimp *et al*. (2004)
*Primula elatior*	Herb.	Neutral	*z*(*r*)	Diversity	Jacquemyn, Brys & Hermy (2002); Jacquemyn *et al*. (2004)
*Ranunculus acris*	Herb.	Neutral	*z*(*r*)	Diversity	Odat, Jetschke & Hellwig (2004)
*Trillium grandiflorum*	Herb.	Neutral	*z*(*r*)	Diversity	Vellend (2004)
*Zostera marina*	Herb.	Neutral	*z*(*r*)	Ecosystem	Reynolds, McGlathery & Waycott (2012)
*Zostera marina*	Herb.	Adaptive	*z*(*r*)	Ecosystem	Hughes & Stachowicz (2009)

### Adaptive genetic diversity

**Figure 1:**
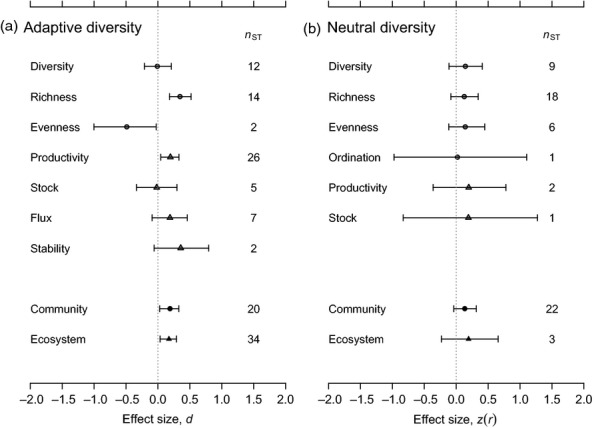
Pooled effects summarizing the variation of community diversity, structure and ecosystem functioning with measures of (a) adaptive genotypic diversity and (b) with neutral molecular genetic diversity. Circular symbols indicate community-level responses, and triangular symbols indicate ecosystem-level responses. The community-level outcome types (species diversity, species richness, species evenness and ordination) and the ecosystem-level outcome types (productivity, stock, flux, stability) are defined in Table 1. The entries labelled ‘community’ and ‘ecosystem’ summarize the effects shown in the upper portion of each plot. Point estimates are posterior mean values. *n*_ST_ gives the number of studies (cf. effect sizes) underpinning each pooled effect. Positive values indicate that community and ecosystem responses increase with increasing genetic diversity. Error bars show 95% credible intervals.

Both community- and ecosystem-level responses to adaptive genetic diversity were consistently positive (*d *=* *0.189 and 0.173, respectively; Fig. 1a); effect sizes for these components of ecological structure did not differ significantly (Fig. 1a). Adaptive community-genetic effect sizes also varied between different types of ecological response measure. For example, species richness and productivity were consistently greater in the presence of more genetically diverse focal species’ populations (*d *=* *0.346 and 0.195, respectively; Fig. 1a). In contrast, species evenness was lower in communities containing more genetically diverse focal species’ populations (although this result was supported by effect sizes from only two studies; Fig. 1a). Effect sizes for species diversity, stocks and fluxes of elements and nutrients and ecosystem stability were not consistently different from zero (effect sizes for fluxes and stability were positive, but were not significantly so; Fig. 1a).

**Figure 2:**
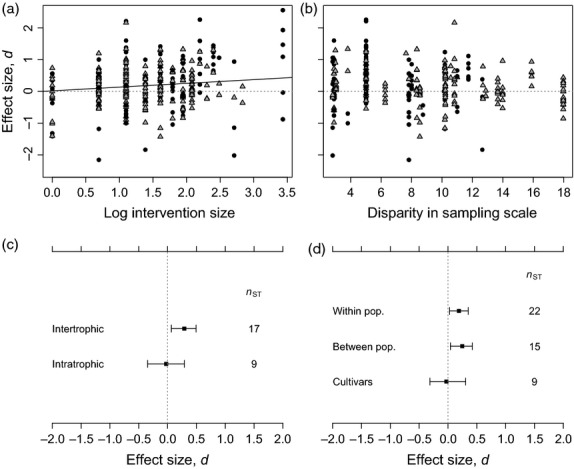
Variation of community-genetic effect sizes with sources of heterogeneity for studies that manipulated adaptive genetic diversity (genotypic richness) experimentally. Variation of effect sizes with (a) the size of the genotypic richness manipulation (intervention size), (b) the disparity between the spatial scales at which genotypes were collected and at which experiments were carried out, and between (c) experiments observing ecological responses within either the same, or a different trophic level to the focal plant species, and (d) different sampling strategies for observing the focal species’ populations and associated ecological effects. ‘Within pop.’ and ‘between pop.’ refer to within- and between-population sampling strategies, respectively. Points in panels (a) and (b) show individual effect sizes (without their corresponding measurement error variance); symbols are as described in Fig. 1. Points and error bars in the remaining plots follow the definitions given in Fig. 1.

Larger ecological effects of genetic diversity were observed when effect sizes compared focal populations with a greater difference in adaptive genetic diversity (*pMCMC* = 0.012; Fig. 2a). This result shows that the quantity of genetic diversity, and not simply the presence of genetic diversity *per se*, is important in mediating community and ecosystem-level diversity, structure and function. Effect sizes describing community-level responses were significantly greater than zero only for inter-trophic contrasts, that is, where the responding (dependent) community was at a different trophic level than the focal plant population (Fig. 2c). Intra-trophic effect sizes, describing interactions between focal plant populations and the associated plant community, were not consistently different from zero. In studies that manipulated genetic diversity, effect sizes were not significantly predicted by the mismatch in sampling scale between the focal plant collection sites and the sizes of experimental plots used (Fig. 2b). However, there was an apparent trend in the variance of effect sizes; studies with more closely matched sampling and experimental spatial scales had more variable effect sizes (Fig. 2b). There was no relationship between measurement error variance and the disparity in sample scales, indicating that the increased variance of effect sizes for studies with well-matched spatial designs was not due to larger *mev*, that is, noise. The studies with greatest disparity in sample scales (and least variable effect sizes) used *Arabidopsis thaliana* as the focal plant species. Adaptive community-genetic effect sizes were positive both for studies that sampled focal experimental individuals from within a single population or for studies that drew focal individuals from a number of different populations (Fig. 2d). However, studies that used agricultural cultivars to form focal plant populations showed lower effect sizes that did not differ significantly from zero (Fig. 2d).

### Neutral genetic diversity

**Figure 3:**
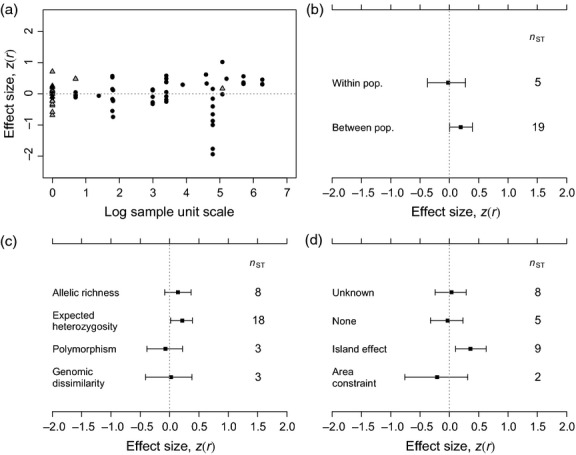
Variation in community-genetic effect sizes based on neutral genetic diversity with different sources of heterogeneity. Variation of effect sizes with (a) the spatial extent of the sampling units within which genetic diversity was measured, (b) sampling strategy for focal species sampling units, that is, whether these were within or between populations, and between (c) types of genetic diversity recorded for the focal plant species, and (d) locality characteristics that may influence the relationship between genetic diversity and measures of ecological structure (see methods for definitions of terms). Symbols, points and error bars follow descriptions in Fig. 1.

Neutral genetic diversity showed no overall consistent association with ecological structure at either the community or ecosystem levels, or for any component type of ecological response (Fig. 1b). However, positive relationships between genetic diversity and ecological structure were observed in cases where the expected heterozygosity had been used as a measure of genetic diversity and for studies that sampled spatially discrete populations of the focal plant species (*z*(*r*) = 0.232 and *z*(*r*) = 0.243, respectively; Fig. 3). The largest effect sizes were observed in situations where populations of the focal species both varied in spatial extent and were isolated from each other as effective demographic islands. These cases showed consistently positive genetic diversity–ecological structure relationships (*z*(*r*) = 0.358; Fig. 3).

### Heterogeneity and publication bias

Variation in effect sizes among studies that manipulated adaptive variation accounted for between 29.2 and 37.0% of the total heterogeneity in effect sizes (Table 3). Effect sizes within studies were consistent in magnitude (heterogeneity among effect sizes within studies was less than 8% of the total). Measurement error variance (within-effect size variance) accounted for the remaining 57.7–67.7% of heterogeneity. Studies documenting community-genetic effect sizes using correlative approaches applied to neutral genetic diversity showed a distribution of heterogeneity more strongly skewed towards between-study variance (52.3–63.3% between-study; 1.5–2.1% between effect sizes within study; 35.1–45.8% within effect size; Table 3). Results from meta-analyses can be biased if the studies that were published (and reviewed) were a biased set of the total studies that were undertaken by researchers. If there had been publication bias towards studies showing a positive relationship between genetic diversity and measures of ecological structure, then we would expect to see a deficit of statistically insignificant negative effect sizes (i.e. negative effect sizes with large measurement error variance or standard error). Forest plots of effect size against effect size standard error showed little evidence for asymmetry in the distribution of effect size estimates that would indicate the presence of publication bias (Fig. S1).

**Table 3:** Summary of models fitted to community-genetic effect size data. Study and residual variance give point estimates for variance components (variance in effect sizes among studies and between effect sizes within studies, respectively, after conditioning on the fixed effects). The percentage of heterogeneity that these effects account for is shown in parentheses. The remaining heterogeneity (100% – study variance% – residual variance%) is attributable to variation within effect sizes (i.e. the measurement error variance). The final three columns give model-checking statistics: the minimum number of effective samples across all parameters (values closer to 1000 indicate a lower degree of autocorrelation within MCMC samples), the maximum absolute autocorrelation of MCMC samples at lag 1 across all parameters (values closer to 0 indicate a lower degree of autocorrelation within MCMC samples) and the maximum potential scale reduction factor across all parameters (PSRF; Gelman–Rubin diagnostic; values closer to 1 indicate better convergence of replicate MCMC chains)

Effect size measure*	Fixed effects†	Study variance (%)	Residual variance (%)	Effective samples	Auto-correlation	PSRF
*d*	∼Intercept	0.118 (35.5)	0.018 (5.3)	1000	0.044	1.010
*d*	∼Outcome level (community/ecosystem)	0.119 (35.6)	0.018 (5.4)	880	0.063	1.007
*d*	∼Outcome type	0.085 (29.2)	0.009 (3.1)	711	0.090	1.010
*d*	∼Intervention size	0.115 (35.2)	0.016 (4.9)	1000	0.041	1.009
*d*	∼Trophic contrast	0.180 (32.8)	0.035 (6.4)	769	0.039	1.010
*d*	∼Sampling strategy	0.126 (37.0)	0.018 (5.2)	717	0.055	1.007
*d*	∼Disparity in sampling scale	0.111 (33.8)	0.026 (7.9)	960	0.037	1.006
*z*(*r*)	∼Intercept	0.120 (58.1)	0.003 (1.6)	947	0.037	1.007
*z*(*r*)	∼Outcome level (community/ecosystem)	0.131 (60.3)	0.003 (1.5)	1000	0.049	1.010
*z*(*r*)	∼Outcome type	0.150 (63.3)	0.004 (1.5)	664	0.061	1.005
*z*(*r*)	∼Sample unit scale	0.098 (53.0)	0.004 (2.0)	873	0.067	1.010
*z*(*r*)	∼Sampling strategy	0.100 (53.6)	0.004 (1.9)	1000	0.019	1.007
*z*(*r*)	∼Genetic diversity type	0.096 (52.5)	0.004 (2.1)	704	0.034	1.006
*z*(*r*)	∼Locality characteristics	0.091 (51.1)	0.004 (2.1)	828	0.049	1.007

* Effect sizes were either standardized mean differences (*d*) or *z*-transformed correlation coefficients (*z*(*r*)).

† Sources of heterogeneity fitted as effects in the meta-analytic models.

## Discussion

In this review, I carried out a meta-analysis assessing the relationship between genetic diversity within plant populations and the structure and functioning of associated communities and ecosystems. These analyses were novel in allowing an assessment of the strength and direction of the relationship between genetic diversity and ecological structure. They also enabled a first comparison of ecological effects associated with adaptive and neutral genetic diversity. My results show that ecological responses stemming from adaptive genetic diversity are significantly positive at both community and ecosystem levels of organization. In contrast, the pooled effect size for neutral genetic diversity did not differ consistently from zero. However, these latter effect sizes were also consistently positive under certain demographic conditions.

I found that the positive association between adaptive genetic diversity and community-level structure was driven by genetic effects on species richness (Fig. 1a). Neither species diversity (e.g. Shannon diversity) nor evenness reacted in a similar way (Fig. 1a), suggesting that adaptive genetic diversity does not have a consistent impact on all components of community diversity. I also found that effect sizes for adaptive genetic diversity were positive only for ‘inter-trophic’ experiments, which compared the genetic diversity of focal plant populations to ecological responses occurring at other trophic levels (Fig. 2). Collectively, these results suggest that genetically controlled phenotypic changes within focal plant populations drive differences in the richness, but not the diversity or evenness of the communities that depend on them. This relationship may be explained by a positive effect of plant genetic diversity on plant population productivity (Fig. 1a; Crutsinger *et al*. 2006; Cook-Patton *et al*. 2011). The greater productivity of genetically diverse plant populations could increase the quantity of biomass available as a habitat for dependent species, allowing the richness of these species to accumulate as a function of increases in total abundance (Srivastava & Lawton 1998). However, specialization of the dependent species on different plant genotypes could also be important in enhancing their species richness on genetically diverse plant populations (Hutchinson 1959). Both of these mechanisms have been found to contribute to the richness of arthropod communities colonizing experimental populations of *Solidago altissima* (Crutsinger *et al*. 2006).

The adaptive diversity of plant populations was not a significant predictor of the diversity and structure of the plant communities of which they were a component part (‘intra-trophic’ studies; Fig. 2). In these systems, coexisting and potentially co-dominant plant species may have an effect on community structure or ecosystem functioning that is at least as strong as the effects of genetic diversity within any one component species. For example, biomass production in model sand dune plant communities was determined by an interaction between initial species richness within the communities and genotypic diversity within one of the constituent species (*Ammophila breviligulata*; Crawford & Rudgers 2012). In limestone grassland communities, genotypic diversity can have weak effects on species diversity, but these effects are environmentally contingent (Fridley & Grime 2010) and can take a long time to develop (Booth & Grime 2003). Thus, the ecological effects of genetic diversity were weaker within communities occupying a single trophic level than they were for systems where dependent communities respond to a dominant foundation species. This difference in effect sizes was predicted in each of two narrative reviews (Johnson & Stinchcombe 2007; Hughes *et al*. 2008).

The component of ecosystem functioning most strongly associated with adaptive genetic diversity was biomass productivity (Fig. 1a). For example, genetically diverse populations of *Arabidopsis thaliana* produced a greater quantity of biomass than genetically impoverished single-ecotype populations (Crawford & Whitney 2010). Other elements of ecosystem functioning, such as stocks and fluxes of nutrients and elements, were not significantly predicted by genetic diversity. These results are likely to be underpinned by the fact that plant productivity is a direct phenotypic expression of the plant genotype, whereas other aspects of ecosystem functioning are indirectly or more weakly connected with causal genotypes (Bailey *et al*. 2009).

My results demonstrated an ecological effect of adaptive diversity *per se* (Fig. 1), but also an additional effect of increasing genetic diversity (Fig. 2a), suggesting that ecological structure continues to be modified as genotypic diversity is accumulated within focal species. This finding begs the question of whether conservation practitioners should seek to deliberately enhance genotypic or adaptive diversity in restoration or translocation projects (Vergeer, Sonderen & Ouborg 2004; Reynolds, McGlathery & Waycott 2012) in order, for example, to boost species richness in associated arthropod communities (Bangert *et al*. 2005; Whitham *et al*. 2006). The results presented in this review do support the idea that management to avoid low levels of genetic diversity in foundation species could bring benefits to their productivity and to the species richness of dependent communities (e.g. Crutsinger *et al*. 2006; Cook-Patton *et al*. 2011; McArt, Cook-Patton & Thaler 2012). However, avoidance of the very low levels of genotypic diversity typically investigated in the community genetics literature (e.g. monoclonal populations) is likely to be easy to achieve and may not require alteration of best practice in conservation management. In addition, our understanding of these effects is generally limited to fine spatial scales (e.g. sample plots ≤ 1 m^2^ in size; Reusch *et al*. 2005; Vellend, Drummond & Tomimatsu 2010) and, in ecological terms, to short time frames (e.g. studies within a single field season or within a single clonal generation; Johnson, Lajeunesse & Agrawal 2006; Fridley & Grime 2010). Furthermore, any benefits to species richness could be accompanied by an equivalent cost to species evenness (Fig. 1a), which may undermine the initial gain in richness and the longer-term sustainability of dependent communities. For example, McArt, Cook-Patton & Thaler (2012) observed that genotypic mixtures of *Oenothera biennis* selected for arthropod communities had greater species richness, but lower evenness and diversity than genotypic monocultures. The greater richness of arthropod species was attributed to a greater total resource for arthropods living on genotypic mixtures, while the decrease in diversity and evenness was ascribed to the disproportionate increase in abundance of a dominant arthropod species (*Plagiognathus politus*) on genotypic mixtures, relative to monocultures. Other studies focussing on *Oenothera biennis* have shown that both selective impacts of arthropods on the foundation plant population and corresponding selection flowing from the plant to the dependent arthropod community are able to occur through time (Agrawal *et al*. 2012, 2013). Therefore, modifications to arthropod community structure (evenness) could result in a selective feedback to the focal plant population that subsequently destabilizes initial benefits to arthropod species richness.

The message for conservation is that we still know too little regarding the impacts of genetic diversity on ecological structure to recommend management for high genetic diversity as a means to enhance the diversity or functioning of ecosystems. However, the results presented in this review do strengthen the growing consensus that careful conservation management for the maintenance of genetic diversity (managing for ‘genetic health’) can lead to greater sustainability in populations and communities that are the focus of conservation efforts. Such management may lead to avoidance of the deleterious consequences of inbreeding (Hedrick & Kalinowski 2000), maximization of evolutionary potential (Neaves *et al*. 2013) and avoidance of costs arising from outbreeding between genetically divergent populations (Whitlock *et al*. 2013).

Pooled community-genetic effect sizes for adaptive diversity at the community and ecosystem levels were *d *=* *0.189 and *d *=* *0.173, respectively, and qualify as ‘small’ under Cohen's (1988) classification of effect sizes. These effect sizes are also small in comparison with average effect sizes observed more widely in the evolutionary ecology literature (range in average effect sizes (*d*), 0.631–0.721; Møller & Jennions 2002). Furthermore, the pooled effects observed in this study were substantially lower in magnitude than comparable effects reported by Bailey *et al*. (2009). These authors reported a pooled effect size for the community-level effects of adaptive genotypic diversity of *d *=* *0.464 (following conversion from *z*(*r*) to *d*; Borenstein *et al*. 2009), compared with *d *=* *0.189 in this study (Fig. 1). These differences in effect size magnitude seem very likely to have occurred because Bailey *et al*. (2009) transformed all effect sizes so that they had a positive value (absolute transformation), which would have introduced an upward bias in the pooled effects. For example, if the effect sizes in my data set for adaptive diversity are transformed in the same way, then the effect size for community-level responses changes from 0.189 to 0.581.

A further difference between my meta-analysis and that of Bailey *et al*. (2009) was evident in the scale of confidence intervals (or credible intervals) surrounding pooled effects. A likely explanation for this disparity lies in my use of study-level random effects to take into account the nesting of effect sizes within studies. Study-level heterogeneity in effect sizes accounted for up to 37.0% (adaptive genetic diversity) or 63.3% (neutral genetic diversity) of the total heterogeneity present, and effect sizes within studies showed a marked consistency (Table 3). Refitting the meta-analysis without study random effects (i.e. running the analysis as a ‘traditional’ random-effects meta-analysis; Viechtbauer 2010) gives *d *=* *0.307, 95% confidence interval = 0.210–0.404, as the pooled effect for adaptive community-level effect sizes (note the narrower credible interval cf. Fig. 1.) In summary, my results show that ecological responses to adaptive genetic diversity are smaller and less predictable than the previous analysis had suggested.

Between-study variation in community-genetic effect sizes may be attributable to unmeasured biological variables, such as study species life history or demographic structure, or choice of study population, or to differences in methodological approaches taken by different authors or research groups. This heterogeneity may also arise because it is extremely difficult to predict how the effects of intraspecific genetic diversity will influence associated species that are either highly dominant within their trophic level or are ecosystem engineers and that modify the environment for a dependent community. For example, greater levels of plot-level genotypic diversity in *Solidago altissima* increased the abundance of galls formed by the midge *Rhopalomyia solidaginis* (Crawford, Crutsinger & Sanders 2007), and presence of the galls led to increases in arthropod richness. In contrast, increased genotypic richness in *Oenothera biennis* led to a less even species abundance distribution in the community of dependent arthropods because of a disproportionate increase in the abundance of a single dominant arthropod species (*Plagiognathus politus*; McArt, Cook-Patton & Thaler 2012). In short, part of the unpredictability of the responses of dependent communities to genetic diversity within a foundation species arises because it is difficult to anticipate precisely how ecological interactions within associated or dependent communities will play out.

A key objective of this review was to determine whether neutral and adaptive measures of genetic diversity have comparable relationships with ecological structure. In contrast to adaptive genetic diversity, neutral genetic diversity showed no overall significant association with ecological structure (Fig. 1). Unlike adaptive genetic diversity, neutral genetic variation cannot drive changes in community structure and functioning via the phenotypes of constituent individuals. In spite of this, however, neutral genetic diversity and ecological structure were correlated under certain circumstances. A significantly positive relationship was observed for studies involving populations and communities that represented islands from a demographic perspective and for which there was between-deme (and between-community) variation in spatial extent (‘island effect’ studies; Fig. 3d). Examples of typical study systems included understorey herb communities in forest fragments (total extent of study ≈ 36 km; Vellend 2004) and small calcareous grassland fragments isolated by afforested and urbanized areas (total extent of study ≈ 5 km; Honnay *et al*. 2006). Several studies undertaken on oceanic islands that would have contributed strongly positive effect sizes had to be excluded, because it was unclear whether their sampling designs met the inclusion criteria for this review (Wendel & Percy 1990; Borgen 1996). The positive association observed for the ‘island effect’ studies is consistent with Vellend's prediction that neutral genetic diversity and community diversity should respond in parallel to differences in the spatial extent of habitat patches (Vellend 2003; Vellend & Geber 2005).

Neutral community-genetic effect sizes were also significantly positive where studies used a between-population (and between-community) sampling design (median plot size = 120 m, median total study extent = 36 km; Fig. 3). Corresponding effects in studies investigating plots or patches within populations and communities did not differ from zero (median plot size = 2 m, median total study extent = 100 m; Fig. 3). This difference is likely to share common mechanisms with the ‘island effect’ described above. The relative scale of dispersal of both genes and species in the within-population studies is likely to outstrip the ability of genetic and ecological drift to impose correlation between genetic and species diversity within sampling units. The between-population studies include all of the cases in which communities and populations were identified as having the potential to exist as demographic islands.

Taken together, my results indicate that the measures of adaptive and neutral genetic variation currently used in community genetics studies should not be viewed as ecologically interchangeable. Hence, studies that utilize neutral genetic diversity should not attempt to make a general test of relationships between genetic diversity and ecological structure (cf. Silvertown, Biss & Freeland 2009; Taberlet *et al*. 2012). These studies are only likely to demonstrate positive effects when the demographic conditions stated above are met. Similarly, positive ecological responses to adaptive genetic diversity are not a foregone conclusion; responses will vary depending of the type of ecological response in focus (Fig. 1). At this point, it is important to note that studies that consider neutral genetic diversity often use a different design to those that employ adaptive genetic diversity. The former frequently use an observational approach applied to different naturally occurring populations, while the latter usually use an experimental approach (but see Hughes & Stachowicz 2009; Silvertown, Biss & Freeland 2009; Nestmann *et al*. 2011). It is possible that surveys of adaptive genetic diversity that followed an observational design would detect relationships with ecological structure that are comparable with those reported here for neutral genetic diversity. Results from the very few studies that have taken this approach are consistent with this possibility (Hughes & Stachowicz 2009; Chang & Smith 2012, 2013). However, these studies have yet to document the extent of genetically controlled phenotypic differentiation between clonal individuals observed in the field. To close this knowledge gap, additional studies are needed that survey levels of genotypic variation or genetic variance across natural populations in the field (e.g. Hughes & Stachowicz 2009). In the meantime, I suggest that conclusions drawn from community genetics studies should be carefully limited on the basis of the scope and context of the study, including the type of genetic diversity investigated and the relevant mechanisms at play (Box Box 1; Vellend & Geber 2005; Hughes *et al*. 2008).

The results presented in this review enhance our understanding of when and how genetic diversity and measures of ecological structure are likely to be associated with each other. However, the review also suggests several opportunities for further empirical studies targeted at areas where the evidence base is currently more limited. First, as noted above, we need more studies that assess the relationship between adaptive genetic diversity and ecological structure in non-experimental settings. Secondly, studies that investigated the ecological consequences of adaptive genetic diversity were usually conducted over relatively short time frames that minimized or excluded the development of eco-evolutionary dynamics (e.g. within a single field season or clonal generation) and frequently used measures of genotypic or ecotypic richness, rather than measures of genetic variance. These two design biases could be addressed by the application of carefully designed experiments that incorporate different measures of adaptive genetic variation (as proposed by Hughes *et al*. 2008) or that include a temporal component allowing the development of eco-evolutionary dynamics (e.g. Agrawal *et al*. 2012).

## Conclusions

The meta-analyses presented in this paper provide an objective and quantitative assessment of the relationship between population-level genetic diversity and community structure and ecological functioning. The results illustrate how this relationship varies between the two types of genetic diversity commonly used in community genetics studies among different ecological response measures and in different ecological and spatial contexts. In several instances, these findings bear out predictions and mechanistic interpretations that have been set out in previous narrative reviews (Vellend & Geber 2005; Johnson & Stinchcombe 2007; Hughes *et al*. 2008). My work also offers a benchmark for future studies, by providing estimates of the ecological effect size of genetic diversity in these different contexts. This synthesis moves the field away from binary assessments of the ecological importance of genetics to a position where we can begin to appreciate and document the particular situations in which intraspecific diversity is linked with higher-order ecological structure. It also illustrates the need for an increased focus on the mechanisms underpinning the ecological effects of genetic diversity. Both of these developments are necessary for an effective coupling between community ecology and evolutionary biology (Johnson & Stinchcombe 2007).

## Data accessibility

Community-genetic effect size data sets and sources of heterogeneity (predictor variables) available from the Dryad repository (Whitlock 2014).

## Supporting Information

**Data S1.** Extended definitions for adaptive and neutral genetic diversity. **Data S2.** Supplementary methods for the literature search. **Data S3.** Additional methods for article assessment. **Table S1.** Search terms used to locate relevant literature. **Table S2.** Search terms used to exclude irrelevant medical literature within Endnote. **Figure S1.** Enhanced funnel plots for community-genetic effect sizes.
