# Supplementary material for: Relationships between adaptive and neutral genetic diversity and ecological structure and functioning: a meta-analysis
Source: J Ecol. 2014 Jun 23;102(4):857–72. doi: 10.1111/1365-2745.12240 (PMC4142011; doi:10.1111/1365-2745.12240)
Supplement: Supplementary file 1 — Data S1. Extended definitions for adaptive and neutral genetic diversity. Data S2. Supplementary methods for the literature search. Data S3. Additional methods for article assessment. Table S1. Search terms used to locate relevant literature. Table S2. Search terms used to exclude irrelevant medical literature within Endnote. Figure S1. Enhanced funnel plots for community-genetic effect sizes. [file jec0102-0857-sd1.docx]

**Supporting information**

**Data S1** Extended definitions for adaptive and neutral genetic diversity

Adaptive genetic variants influence the phenotype by modifying either the quantity or structure of expressed proteins, or the timing of their expression. Adaptive diversity can be measured by quantifying the phenotypic effects of DNA sequence variation within particular genomic regions, or at individual gene loci ([e.g. Bergelson & Roux 2010](#_ENREF_1)). However, in ecological studies, the presence of adaptive variation is more often inferred by observing the phenotypes of individuals under a common environment ([e.g. Tack & Roslin 2011](#_ENREF_5)). These studies usually include both related and unrelated related individuals; offspring plants derived from the sexually produced seed of a single maternal individual must be related as either half sibs or full sibs; plants derived clonally from a single maternal individual are genetically identical (i.e. part of the same genet). If related individuals possess more similar phenotypes than unrelated individuals, then there is a genetic component to the phenotype. The most commonly used measure of adaptive diversity in the community genetics literature is clonal or ecotypic richness (the number of genetically distinct clones or ecotypes in a population). However, adaptive genetic variation can also be measured as broad- or narrow-sense genetic variance, and arguably, these are more appropriate measures, because they quantify the extent to which genes influence the phenotype ([Lynch & Walsh 1998](#_ENREF_4)). Plant ecologists often refer to the genetically distinct individuals of clonal plant species as “genotypes”, and I follow this convention here (cf. population genetics, where a genotype is a description of the allelic state at a single genetic locus). It should be noted that some of the phenotypic variation contained within populations could be selectively neutral under the prevailing environment. In this paper such phenotypic variation is treated as “adaptive”, as long as there is evidence that this variation possesses an underlying genetic component.

Neutral genetic diversity is comprised of sequence variation that does not influence the phenotype, and includes synonymous nucleotide substitutions within genes, or any sequence variation outside of genes and their regulatory regions. Neutral genetic variation is often exploited as a source of molecular markers for genotyping studies. For example, amplified fragment length polymorphism markers (AFLP) and microsatellite markers depend upon sequence variation that is expected to be selectively neutral. These, and other types of DNA markers represent the only tools for directly measuring neutral genetic diversity. Molecular markers can also be used to establish the genetic distinctness of individuals collected from the field ([e.g. Hughes & Stachowicz 2004](#_ENREF_3)). Hence, there is a role for molecular markers in defining adaptive diversity in common garden experiments, because individuals of clonal organisms must be demonstrably genetically distinct before among-clone differences in phenotype can be attributed to genetic causes. Although most marker loci will behave in a neutral fashion, a small subset of marker loci may be in linkage disequilibrium with adaptive genetic variation (e.g. gene loci), and these may show associations with particular phenotypes or selective environments ([Bonin *et al.* 2007](#_ENREF_2)).

**Table S1.** Search terms used to locate relevant literature. Syntax follows the Web of Science standard. Search strings were combined using Boolean operators as follows: ((1) AND ((2) OR (3))) OR (4)

| **String** # | **Target subjects** | **Search strings** |
| --- | --- | --- |
| (1) | Genetic diversity | genet* OR genot* OR "intraspecific diversity" OR "intraspecific vari*" |
| (2) | Community structure and diversity | "community structure" OR "species diversity" OR "species richness" OR "species composition" OR "alpha diversity" OR "species evenness" OR "species abundance" OR "community diversity" OR "community function*" OR (community AND resistance) OR (community AND resilience) OR (community AND succession) |
| (3) | Ecosystem function | "ecosystem function*" OR "ecosystem process*" OR (("primary production" OR "primary productivity" OR productivity OR biomass OR *minerali?ation OR leach* OR fixation OR "denitrification" OR respiration OR "nutrient cyc*" OR decompos* OR flux OR uptake OR precipitation OR litter OR exudate OR resistance OR resilience) AND Ecosystem*) |
| (4) | Catch-all | "community genetic*" |

**Data S2**. Supplementary methods for the literature search

All literature searches interrogated the title, abstract and keywords of articles within databases, except for the Scopus searches, which were limited to title and abstract only. Results from Science Direct and Scopus searches were limited to journal articles. The Endnote database was searched for irrelevant clinical and biomedical articles, resulting in the exclusion of 1289 articles. In order to check the validity of this procedure, I assessed a random subset of 100 of these articles using the review scope and inclusion criteria given below. No relevant articles were detected. A further 401 non-journal articles were excluded from the Endnote database leaving a total of 6980 articles that were assessed for relevance against the aims and scope of the review.

**Table S2.** Search terms used to exclude irrelevant medical literature within Endnote. Each search for irrelevant material was independent.

| **Term** | **Scope** |
| --- | --- |
| Clinical | All fields |
| “Health care” | All fields |
| Healthcare | All fields |
| AIDS | Journal title only |
| Medic | Journal title only |
| Children | All fields |
| Hospital | All fields |
| Psych | All fields |

**Data S3** Additional methods for article assessment

Articles titles were assessed for presence of the correct article type (empirical reports and relevant reviews). Assessment of the remaining criteria within the review scope was simplified to an appraisal of whether the title suggested the presence of within species variation and any other level of diversity, structure or functioning (e.g. species-level, community, habitat or ecosystem-level). Titles were scored as potentially relevant where this assessment was ambiguous. Thus I accepted the possibility that non-relevant articles would be included as potentially relevant at this stage.

1153 articles passed title assessment, and I read the abstracts of these articles and assessed them for relevance as follows. For each abstract I determined the presence of relevant subjects, correct exposure variable (genetic diversity) and relevant outcomes. Where it was clear from the abstract text that a study lacked one or more of these components it was scored as irrelevant. I categorized the experimental design of the remaining relevant studies in order to identify potentially relevant studies, and map the structure of the literature.

1. Studies that included observations on experimental or natural populations that differed in their level of genetic diversity (genotypic, quantitative or molecular), were scored as relevant to this review. This category included, for example, studies that created experimental populations or communities that differed in the number of genetically distinct individuals that they contained, and surveys of natural populations that documented both genetic diversity and community structure or ecosystem function. Studies were retained as potentially relevant in cases where valid subjects, correct exposure variable and relevant outcomes were present, but it was not clear from the abstract whether exposure and outcomes had been compared directly. Articles were also retained in cases where it was not clear whether community-level outcome measures were multi-species in nature or not.
2. Studies that made observations on relevant ecological outcomes separately for each distinct genetic individual, genotype or genetic lineage (i.e. no population-level genetic diversity) were noted, but were scored as non-relevant. Such studies provide evidence for variation in ecological outcome variables within a population of a focal species. Examples include studies using common garden designs to observe community structure or ecosystem-level effects of individual clones, or hybrid classes. Studies that focused on GM crop or livestock cultivars versus non-GM parental lineages also belonged in this category. Quantitative genetics studies documenting the genetic architecture of the relevant outcome measures within a single focal species’ population also qualified as non-relevant under this category (e.g. direct and indirect genetic effects on ecological outcome variables, genetic correlations describing the trade-offs among outcome measures).
3. The final category contained studies in which genotype x genotype interspecific interactions were addressed. The presence of these studies was noted, but they were not counted as relevant for the purposes of the review.

236 articles passed abstract assessment and the fulltexts for these were downloaded and assessed stringently against the full set of review scope criteria. At this stage I identified articles focusing on focal species populations or responding communities or ecosystems that involved plants. These articles were retained as relevant while articles dealing exclusively with animals, microbes or fungi were noted but not used in this review. During fulltext assessment, I identified several studies in which the sampling strategy for selecting individuals for different genetic diversity treatments was both non-random and varied systematically with genetic diversity treatment. These studies were excluded. I also excluded studies providing information on the population-level correlation between genetic diversity and community structure or ecosystem function where these dealt with fewer than five populations.

**Figure S1** Enhanced funnel plots for community genetic effect sizes, implemented using the R package metafor ([Viechtbauer 2010](#_ENREF_6)). If publication bias is present in the community genetics literature then the distribution of effect sizes for small studies (those whose effect sizes have large standard error) should be asymmetrical. For example, a deficit of negative effect sizes would be observed in these small studies if there had been a bias towards a positive relationship between genetic diversity and ecological structure. The plots in the left-hand panels show the full set of effect sizes for the standardised mean difference (*d*) and *z*(r) community-genetic effect size datasets. The right-hand plots show enlargements of the left-hand plots. The vertical line in each plot indicates the pooled effect size for a random-effects model including an intercept as the only fixed effect. Shaded areas of the funnel give pseudo-confidence interval regions: grey shading, 95% pseudo-confidence interval region; dark grey shading, 99% pseudo-confidence interval region. The pseudo-confidence regions incorporate between-effect size heterogeneity.

**References**

Bergelson, J. & Roux, F. (2010) Towards identifying genes underlying ecologically relevant traits in *Arabidopsis thaliana*. *Nature Reviews: Genetics,* **11,** 867–879.

Bonin, A., Ehrich, D. & Manel, S. (2007) Statistical analysis of amplified fragment length polymorphism data: a toolbox for molecular ecologists and evolutionists. *Molecular Ecology,* **16,** 3737–3758.

Hughes, A.R. & Stachowicz, J.J. (2004) Genetic diversity enhances the resistance of a seagrass ecosystem to disturbance. *Proceedings of the National Academy of Sciences of the USA,* **101,** 8998–9002.

Lynch, M. & Walsh, B. (1998) *Genetics and analysis of quantitative traits*. Sinauer Associates, Inc., Sunderland, MA, USA.

Tack, A.J.M. & Roslin, T. (2011) The relative importance of host-plant genetic diversity in structuring the associated herbivore community. *Ecology,* **92,** 1594–1604.

Viechtbauer, W. (2010) Conducting meta-analyses in R with the metafor package. *Journal of Statistical Software,* **36,** 1–48.
